# Secure Chaotic Map Based Block Cryptosystem with Application to Camera Sensor Networks

**DOI:** 10.3390/s110201607

**Published:** 2011-01-27

**Authors:** Xianfeng Guo, Jiashu Zhang, Muhammad Khurram Khan, Khaled Alghathbar

**Affiliations:** 1 Key Laboratory of Signal and Information Processing of Sichuan Province, School of Information Science & Technology, Southwest Jiaotong University, Chengdu, China; E-Mail: jszhang@home.swjtu.edu.cn; 2 College of Computer Science and Technology, Southwest University for Nationalities, Chengdu, China; E-Mail: guoxianf@126.com; 3 Center of Excellence in Information Assurance, King Saud University, Riyadh, Saudi Arabia; E-Mail: mkhurram@ksu.edu.sa; 4 Information Systems Department, College of Computer and Information Sciences, King Saud University, Riyadh, Saudi Arabia; E-Mail: kalghathbar@ksu.edu.sa

**Keywords:** cryptography, camera sensor network, chaotic, key stream attack

## Abstract

Recently, Wang *et al.* presented an efficient logistic map based block encryption system. The encryption system employs feedback ciphertext to achieve plaintext dependence of sub-keys. Unfortunately, we discovered that their scheme is unable to withstand key stream attack. To improve its security, this paper proposes a novel chaotic map based block cryptosystem. At the same time, a secure architecture for camera sensor network is constructed. The network comprises a set of inexpensive camera sensors to capture the images, a sink node equipped with sufficient computation and storage capabilities and a data processing server. The transmission security between the sink node and the server is gained by utilizing the improved cipher. Both theoretical analysis and simulation results indicate that the improved algorithm can overcome the flaws and maintain all the merits of the original cryptosystem. In addition, computational costs and efficiency of the proposed scheme are encouraging for the practical implementation in the real environment as well as camera sensor network.

## 1. Introduction

Camera Sensor Networks (CSNs) are usually built with a large number of inexpensive, small and battery-powered devices. They have been used for a wide variety of applications such as environment monitoring, health monitoring, military sensing and tracking, *etc.* [1]. As CSNs are widely deployed in remote and hostile environments to transmit sensitive information by broadcast, sensor nodes are prone to node compromise attacks and security issues such as data confidentiality and integrity are extremely important. Hence, security becomes a very serious concern in wireless CSN protocols. Unfortunately, the sensors have limited power, computation, storage and communication capabilities, they impose several constraints on the algorithms and protocols that can be effectively deployed for such systems. In this scenario, most of the traditional security mechanisms are useless. Thus, the research of new efficient security techniques such as block and stream cipher [2,3] is needed.

As a very complicated phenomenon of nonlinear system, chaos has inherent analogous cryptographic properties such as sensitive to parameter and initial state, which inspires people to apply it into cryptography [4,5] are representative works. Since Baptista proposed a novel cryptosystem based on the property of ergodicity of chaotic systems [5], a number of new algorithms based on variations of Baptista’s one have been published [6,7]. However, most of those modified methods can’t possess both fast encryption speed and flat ciphertext distribution. To solve these problems, Xiang *et al.* [8] proposed a novel chaotic block cryptosystem based on [5,9,10]. Unfortunately, the sub-keys of this scheme are independent of the plaintext and are determined only by the secret key, which will cause chosen plaintext attack and differential known-plaintext attack [11,12]. Wang *et al.* [11] put forward an improved version by utilizing ciphertext feedback.

This paper studies the security of Wang *et al.* scheme and reports the following findings: (1) Without the secret key, any ciphertext can be decrypted by using only two identical length of chosen ciphertext sequences; (2) It is vulnerable to key stream attack (KSA), *i.e.*, the underlying chaotic key stream sequence of any key (*μ*, *x*_0_) can be deduced from some chosen plaintext and ciphertext pairs. By utilizing the calculated chaotic key stream sequence, any ciphertext encrypted by key (*μ*, *x*_0_) can be decrypted efficiently. To provide an efficient cryptographic primitive and eliminate the weaknesses of Wang *et al.* scheme, this paper presents a modified chaotic block cryptographic algorithm on CSN. Security analysis shows that the proposed scheme is more secure than the original one. In addition, the high computational efficiency promotes its application in CSN.

The rest of this paper is organized as follows. Section 2 briefly reviews the Wang *et al.* scheme. Section 3 elaborates the chosen ciphertext attack (CCA) and the key stream attack (KSA). A secure chaotic block cipher in camera sensor network and its performance analysis are given in Section 4 and 5. Conclusions are drawn in Section 6.

## 2. Review of Wang *et al.* Cryptosystem

In this cryptosystem, the secret key is (*μ*, *x*_0_), where *μ* and *x*_0_ is the initial condition and control parameter of the following chaotic logistic map, respectively:

(1) τ(x)=μx(1−x),  x∈[0,1]

Writing the value of *x* in a binary representation:

(2) $$x=0.b_{1}(x)b_{2}(x)\cdots b_{i}(x)\cdots ,x\in [0,1],b_{i}(x)\in \{0,1\}.$$

**Figure 1.**
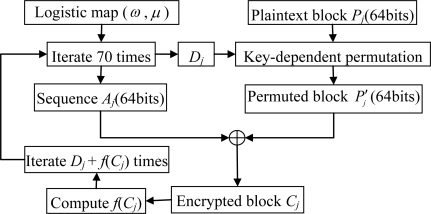
Block diagram of Wang *et al.* scheme.

A binary sequence $B_{i}^{n}={\left\{b_{i}(\tau^{n}(x))\right\}}_{n=0}^{\infty }$, where *n* is the length of the sequence and *τ^n^* (*x*) is the *n*th iteration of the logistic map, can be obtained by iterating the logistic map. The whole procedure of this scheme can be described in the following steps and an illustration is given in Figure 1.

Step 1. Get the start point *ω* which denotes the real value of *x* from the last *N*_0_ transient iterations, *i.e.*, *ω* = *τ^N^*^_0_^ (*x*_0_). Note that we set *N*_0_ = 100 in all the following simulations.
Step 2. Divide the plaintext *P* into subsequences *P_j_* of length *l* bytes (here *l* = 8):
  (3) $$P=P_{1}P_{2}\cdots P_{j}\cdots$$
Step 3. Set *j* = 1;
Step 4. Based on the method to generate binary sequences by iterating the logistic map, obtain a 64-bit binary sequence $A_{j}=B_{i}^{1}B_{i}^{2}\cdots B_{i}^{64}$ and a 6-bit binary sequence $A_{j}^{\prime }=B_{i}^{65}B_{i}^{66}\cdots B_{i}^{70}$ formed by all the third bits, *i.e.*, *i* = 3 in Equation (2), through 70 iterations of the logistic map. *D_j_* is the decimal value of $A_{j}^{\prime }$.
Step 5. Compute the *j*th ciphertext block:
  (4) $$C_{j}=(P_{j}<<<D_{j})⊕A_{j}$$
  where <<< and ⊕ denote the left cyclic shift and XOR operation, respectively.
Step 6. Dividing the ciphertext block *C_j_* into 8-bit partitions and obtain the ciphertext $c_{j}^{1},c_{j}^{2},\cdots ,c_{j}^{8}$.
Step 7. If all the plaintexts have already been encrypted, the encryption process is finished. Otherwise, calculate:
  (5) $$f(C_{j})=c_{j}^{1}+c_{j}^{2}+\cdots +c_{j}^{8}$$
  (6) $$D_{j}^{*}=D_{j}+f(C_{j})\;\text{mod}\;64$$
  (7) $$\omega =\tau^{70+D_{j}^{*}}(\omega )$$
  (8) j=j+1
  and go to Step 4.

The decryption process is almost the same as the encryption one. Just need to replace Equation (4) with:

(9) $$P_{j}=(C_{j}⊕A_{j})>>>D_{j}$$

where >>> denote the right cyclic shift operation.

## 3. Cryptanalysis of Wang *et al.* Cryptosystem

According to *Kerchoff* ’s principle [13], the cryptanalyst knows exactly the design and working of the cryptosystem under study except the secret key. The general types of cryptanalytic attacks [14] are enumerated as follows, ordered from the hardest type of attack to easiest: ciphertext only attack, known plaintext attack, chosen plaintext attack and chosen ciphertext attack. In each of these four attacks, the objective is to determine the key that was used. It suffices that one of the attacks is feasible to consider an algorithm insecure.

In the following subsections, we will perform a chosen ciphertext attack (CCA) and a key stream attack (KSA) on Wang *et al.* scheme. For convenient illustration, suppose *P* = *P*_1_*P*_2_⋯*P_j_*⋯ and *C* = *C*_1_*C*_2_⋯*C_j_*⋯ are the plaintext and ciphertext pairs, (*μ*, *x*_0_) and *K* = (*A*_1_*D*_1_)(*A*_2_*D*_2_)⋯(*A_j_D_j_*)⋯ denote the corresponding secret key and key stream, respectively.

### 3.1. Chosen Ciphertext Attack

A chosen-ciphertext attack [15] operates under the following model: an adversary is allowed access to plaintext-ciphertext pairs for some number of ciphertexts of his choice, and thereafter attempts to use this information to recover the key (or plaintext corresponding to some new ciphertext).

In the Wang *et al.* scheme, Equations (5–7) indicate that the space of the feedback message is only 64, *i.e.*, once the secret key (*μ*, *x*_0_) is determined, the key stream *D_j+1_* and *A_j+1_* are determined only by the former ciphertext *f*(*C_j_*) mod 64. To illustration this security loophole, we set the secret keys *μ* = 4, *x*_0_ = 0.1777 and decrypt two different ciphertext sequences. They are C1=“EAFA4D22D326D40C2960D4C5E76…” and C2=“F11ED8CA5F72155E8A99683495F…” in hexadecimal format. Each block of *C_j_*, *f*(*C_j_*) mod 64, *D_j_* and *A_j_* are filled into Tables 1 and 2, respectively.

The simulation results indicate that once *μ*, *x*_0_ and all the former ciphertext blocks have equal *f*(*C_j_*)*mod* 64, any ciphertext has identical sub-key *D_j_*_+1_ and *A_j_*_+1_. This loophole is vulnerable to CCA, one of CCA illustration can be played as follows: (they cannot be showed completely).

(1) Let $f_{2}^{j}$ denotes the 6-bit length of *f*(*C_j_*)*mod* 64 in binary representation. For *j* = 1,2,⋯ select two cipher blocks:

(10) $$C_{j}^{1}=\underset{56\mathrm{bits}}{\underset{︸}{0\cdots 0}}\underset{8\mathrm{bits}}{\underset{︸}{11f_{2}^{j}}}$$

(11) $$C_{j}^{2}=\underset{50\mathrm{bits}}{\underset{︸}{0\cdots 0}}\underset{6\mathrm{bits}}{\underset{︸}{f_{2}^{j}}}\underset{8\mathrm{bits}}{\underset{︸}{0\cdots 0}}$$

From Equation (5), it is not difficult to see that:

(12) $$f(C_{j})\equiv f(C_{j}^{1})\equiv f(C_{j}^{2})\;\text{mod}\;64$$

To demonstrate this procedure, we fill the chosen corresponding *C*^1^ and *C*^2^ of a random selected ciphertext *C* = 218A916626 E5DA55… (in hexadecimal format) into Table 3.

(2) Decrypt $C^{1}=C_{1}^{1}C_{2}^{1}\cdots C_{j}^{1}\cdots$ and $C^{2}=C_{1}^{2}C_{2}^{2}\cdots C_{j}^{2}\cdots$ using the same key (*μ*, *x*_0_) of *C* = *C*_1_*C*_2_ ⋯*C_j_* ⋯, then we can get the corresponding plaintext $P^{1}=P_{1}^{1}P_{2}^{1}\cdots P_{j}^{1}\cdots$ and $P^{2}=P_{1}^{2}P_{2}^{2}\cdots P_{j}^{2}\cdots$. From Equations (6) and (12) we can deduce that *C_j_*, $C_{j}^{1}$ and $C_{j}^{2}$ have the identical corresponding sub-keys *D_j_* and *A_j_*.

(3) Calculate $P_{j}^{1}⊕P_{j}^{2}=((C_{j}^{1}⊕A_{j})>>>D_{j})⊕((C_{j}^{2}⊕A_{j})>>>D_{j})=(C_{j}^{1}⊕C_{j}^{2})>>>D_{j}$

From Equations (10) and (11), we can obtain that:

(13) $$C_{j}^{1}⊕C_{j}^{2}=\underset{50\mathrm{bits}}{\underset{︸}{0\cdots 0}}\underset{14\mathrm{bits}}{\underset{︸}{f_{2}^{j}11f_{2}^{j}}}$$

Therefore, we can determine the value of *D_j_* by searching the position of $f_{2}^{j}11f_{2}^{j}$ in $P_{j}^{1}⊕P_{j}^{2}$.

(4) Using Equation (4) and the conquered *D_j_*, we can calculate $A_{j}=(P_{j}^{1}<<<D_{j})⊕C_{j}^{1}$. To demonstrate these procedures, the chosen *C*^1^ and *C*^2^ of Table 3 are decrypted using *μ* = 4, *x*_0_ = 0.1777. The corresponding plaintext blocks and sub-keys are filled into Table 4.

(5) By utilizing *D_j_* and *A_j_*, it is easy to figure out the plaintext

(14) $$P_{j}=(C_{j}⊕A_{j})>>>D_{j}$$

**Figure 2.**
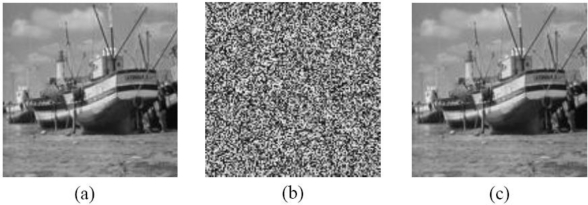
**(a)** Plaintext. **(b)** The ciphertext. **(c)** The result of attack.

Some simulations are utilized to prove the validity of CCA. Figure 2(a–c) are the original image, the encrypted image with Wang et al’s scheme and the analyzed image of a 128 × 128 bitmap image file named Boat, where the secret key *μ* = 4, *x*_0_ = 0.1777 and *N*_0_ = 100.

### 3.2. Key Stream Attack

In the Wang *et al.* scheme [11], although a ciphertext feedback model is employed to ensure sub-keys depend on both secret key and plaintext, a fundamental flaw is unaware, *i.e.*, the first sub-key *D*_1_ and *A*_1_ are independent of the plaintext and are determined only by the secret key (*μ*, *x*_0_). An adversary can reconstruct the key stream sequence as an equivalent key (*μ*, *x*_0_) as follows:

1. Choose two pair of special messages (*P_z_*, *C_z_*) and (*P_s_*, *C_s_*), where *P_z_* is composed of 64-bit zeros, *P_s_* is 011…11 in binary representation, *C_z_* and *C_s_* are the corresponding ciphertext of *P_z_* and *P_s_*, respectively.
2. Set *P_z_* as the first plaintext block, then can get *C_z_* = (*P_z_* <<< *D*_1_) ⊕ *A*_1_ = *A*_1_.
3. Similarly, when set *P_s_* as the first plaintext block, *C_s_* = (*P_s_* <<< *D*_1_) ⊕ *A*_1_, *i.e.*, *C_s_* ⊕ *A*_1_ = *P_s_* <<< *D*_1_. Thus the position of zero in *C_s_* ⊕ *A*_1_ counting from rightmost bit is equal to *D*_1_.
4. Set *k* = 0, and define a plaintext sequence *P_u_* = *ϕ*, where *ϕ* is a null string.
5. *k* = *k* + 1. By utilizing *D_k_*, choose $C_{k}=\underset{56\mathrm{bits}}{\underset{︸}{00\cdots 0}}\underset{8\mathrm{bits}}{\underset{︸}{c_{k}^{8}}}$ to make sure
  (15) $$D_{k}^{*}=D_{k}+f(C_{k})\;\text{mod}\;64=0$$
  From Equation (7), it can be seen that the sub-keys of $A_{k}=B_{i}^{1}B_{i}^{2}\cdots B_{i}^{64}$, $A_{k}^{\prime }=B_{i}^{65}B_{i}^{66}\cdots B_{i}^{70}$, $A_{k+1}=B_{i}^{1}B_{i}^{2}\cdots B_{i}^{64}$ and $A_{k+1}^{\prime }=B_{i}^{65}B_{i}^{66}\cdots B_{i}^{70}$ are continuous state bit of logistic map.
6. Decrypt *C_k_* with *D_k_* and *A_k_*:
  (16) $$P_{k}=(C_{k}⊕A_{k})>>>D_{k}$$
7. Set *P_u_* = *P_u_P_k_*, *i.e.*, add *P_k_* as the last 64 bits of *P_u_*.
8. Encrypt the 64(*k* + 1)-bit length plaintext sequence *P^z^* = *P_u_P_z_*, and then obtain the corresponding ciphertext:
  (17) $$C^{z}=C_{1}^{z}\cdots C_{k}^{z}C_{k+1}^{z}$$
9. Obviously, $C_{1}^{z}\cdots C_{k}^{z}C_{k+1}^{z}$ is equal to *C*_1_ ⋯*C_k_* when *k* > 1, and $C_{k+1}^{z}=(P_{z}<<<D_{k+1})⊕A_{k+1}$. Therefore, it can be calculated that $A_{k+1}=C_{k+1}^{z}$.
10. Encrypt another 64(*k* + 1)-bit length plaintext sequence *P^s^* = *P_u_P_s_*, and then obtain the corresponding ciphertext:
  (18) $$C^{s}=C_{1}^{s}\cdots C_{k}^{s}C_{k+1}^{s}$$
  Similarly, $C_{1}^{s}\cdots C_{k}^{s}$ is equal to *C*_1_ ⋯*C_k_* when *k* > 1, and $C_{k+1}^{s}=(P_{s}<<<D_{k+1})⊕A_{k+1}$, *i.e.*, $C_{k+1}^{s}⊕A_{k+1}=P_{s}<<<D_{k+1}$. Utilizing the computed *A_k_*_+1_, the adversary can obtain *D_k_*_+1_ by counting the position of zero in $C_{k+1}^{s}⊕A_{k+1}$ from rightmost bit.
11. Go to (5) if the length of the key stream sequence is not enough; otherwise, finish the attack.

For *j* = 1,2,⋯,*k*, translate decimal value *D_j_* to the corresponding 6-bit length binary sequence $A_{j}^{\prime }$, and then the adversary can acquire a 70*j*-bit length binary key stream sequence *K* = (*A*_1_*A*_1_′) (*A*_2_*A*_2_′) ⋯ *A_j_A_j_*′ of secret key (*μ*, *x*_0_). We denote *K* = *B*_1_*B*_2_ ⋯ *B*_70_*_j_*.

The key stream *K* can be utilized to decrypt any ciphertext encrypted by (*μ*, *x*_0_). To demonstrate this circumstance, ciphertext *C* = *C*_1_*C*_2_ ⋯ *C_i_* is decrypted as follows:

1. Define *k* = 1. Set the start point of *k*th sub-key in *K* = *B*_1_*B*_2_ ⋯ *B*_70_*_j_* as *n* = 1.
2. Obviously, the *k*th sub-key of *C_k_* is *B_n_B_n_*_+1_ ⋯ *B_n_*_+69_, *i.e.*, *A_k_* = *B_n_B_n_*_+1_ ⋯ *B_n_*_+63_, *D_k_* is the decimal value of *A*′*_k_* = *B_n+_*_64_*B_n_*_+65_ ⋯ *B_n_*_+69_. And then we can obtain the *k*th plaintext block:
  (19) $$P_{k}=(C_{k}⊕A_{k})>>>D_{k}$$
3. If *k* < *i*, continue; otherwise, finish the decryption process.
4. By utilizing the known *C_k_*, *D_k_* and Equations (5) and (6), it is easy to obtain the value of $D_{k}^{*}$. Thus, we can utilize Equation (7) to calculate the start point of (*k*+1)th sub-key in *K* = *B*_1_*B*_2_ ⋯ *B*_70_*_j_*:
  (20) $$n=n+70+D_{k}^{*}$$
5. Compute *k* = *k* +1and go to (2).

As a result, *C* = *C*_1_*C*_2_ ⋯ *C_i_* is decrypted effectively with key stream sequence *K* = *B*_1_*B*_2_ ⋯ *B*_70_*_j_*.

## 4. Proposed Secure Block Cipher for Camera Sensor Networks

### 4.1. Secure Block Cipher Algorithm

The Wang *et al.* cryptosystem is cryptographically weak because information about the feedback value $D_{k}^{*}$ leaks into the ciphertext and the first sub-key is independent of plaintext. Except these flaws, it has some excellent benefits, such as flat ciphertext, fast encryption speed and prominent diffusion and confusion. Therefore it is valuable to propose an improved version to get rid of above flaws. As for the first flaw, it can be remedied via hiding $D_{k}^{*}$ from ciphertext, and the latter can be conquered by pretreating of the first plaintext block. Detail of the improvement is described as follows:

Steps 1–4. They are the same as Wang *et al.* scheme described in Section 2.
Step 5. Compute:
  (21) $$\omega =\tau^{D_{1}}(\omega ),$$
  (22) $$A_{0}=B_{i}^{1}B_{i}^{2}\cdots B_{i}^{64}.$$
Step 6. Obtain the *j*th ciphertext block (*j* ≥ 1):
  (23) $$C_{j}=((P_{j}⊕A_{j-1})<<<D_{j})⊕A_{j}$$
  (24) $$D_{j}^{*}=D_{j}+f(A_{j-1})+f(A_{j}⊕C_{j})\;\text{mod}\;64$$
  (25) $$\omega =\tau^{70+D_{j}^{*}}(\omega )$$

Obviously, after the modified process, the feedback value $D_{j}^{*}$ is hidden from ciphertext. Encrypt *P_z_* and *P_s_*, then one can obtain:

(26) $$C_{s}=((P_{s}⊕A_{0})<<<D_{1})⊕A_{1}$$

(27) $$C_{z}=((P_{z}⊕A_{0})<<<D_{1})⊕A_{1}=(A_{0}<<<D_{1})⊕A_{1}$$

Equations (26) and (27) leak noting about the key stream *A*_1_ and *D*_1_, so the security is enhanced in the improvement. Though it involves some computations, they are not time consuming operations. Therefore, the improved scheme does not lose the original efficiency advantage.

### 4.2. Architecture of Wireless Camera Sensor Networks

**Figure 3.**
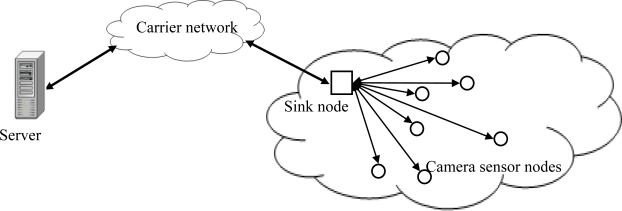
System architecture of the camera sensor network.

In this section, we introduce the developed architecture of the secure wireless camera sensor networks by utilizing the proposed chaotic block cipher. Each camera sensor node in the networks is battery-powered and has limited computation and wireless communication capabilities. The sink is a data collection center equipped with sufficient computation and storage capabilities. Camera sensor nodes periodically send the captured images to the sink node. Then the sink nodes transport this information secretly with the data process server via carrier networks. The proposed block cipher is mounting at the carrier network. Figure 3 shows the system architecture of the camera sensor network.

## 5. Performance Analysis

### 5.1. Information Entropy Analysis

It is known that the entropy *H*(*m*) of a message source m can be calculated by Equation (28) [8]:

(28) $$H(m)=-\sum_{i=0}^{2^{N}-1}p(m_{i})\;\text{log}\;\frac{1}{p(m_{i})}$$

where *p*(*m_i_*) represents the probability of symbol *m_i_*. The entropy is expressed in bits. For a purely random source emitting 2*N* symbols, the entropy is *H*(*m*) = *N*. For encrypted messages, the entropy should ideally be *H*(*m*) = *N* .

When a cipher emits symbols with entropy less than *N*, there exists a certain degree of predictability, which threatens its security. Let us consider the ciphertext of a random text file, a Lena’s image of size 256 × 256 and a random video file encrypted using the proposed scheme. The number of occurrence of each ciphertext pixel *m_i_* is recorded and the probability of occurrence is computed for the three files. The corresponding entropies are filled into Table 5. The test values obtained are very close to the theoretical value *N* = 8 for the three kinds of files. This means that information leakage in the encryption process is negligible and the encryption system is secure against the entropy attack.

### 5.2. Correlation of Adjacent Pixels in Encrypted Image

**Figure 4.**
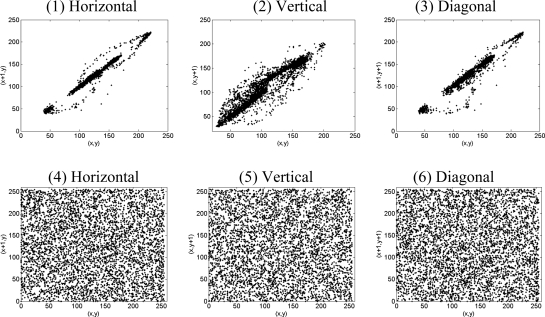
Correlation of the adjacent pixels (1–3)are plaintext and (4–6) are ciphertext.

In order to resist statistical attacks, the ciphertext should possess certain random properties. A detail study has been explored and the results are summarized. The results of the Lena.bmp are used for illustration. For an ordinary image, each pixel is usually highly correlated with its adjacent pixels either in horizontal, vertical or diagonal directions. These high-correlation properties can be quantified as their correlation coefficients for comparison. To calculate the correlation coefficients, the following formulas are used:

(29) $$r(x,y)=\frac{\left|\mathrm{Cov}(x,y)\right|}{\sqrt{D(x)}\sqrt{D(y)}}$$

(30) $$\text{cov}(x,y)=\frac{1}{N}\sum_{k=1}^{N}\left(x_{k}-E(x))(y_{k}-E(y)\right)$$

(31) $$E(x)=\frac{1}{N}\sum_{k=1}^{N}x_{k}$$

(32) $$D(x)=\frac{1}{N}\sum_{k=1}^{N}\left(x_{k}-E(x)\right)$$

where *x* and *y* are the grey-scale value of two adjacent pixels in the image and *N* is the total number of pixels selected from the image for the calculation. In Table 6 and Figure 4, the correlation coefficients of Lena image and those of its encrypted image with the secret key (*μ* = 3.998, *x_0_* = 0.21745) are given.

It is clear that there is negligible correlation between these two adjacent pixels in the encrypted image. However, the two adjacent pixels in the original image are highly correlated. The results indicate that the proposed algorithm has successfully removed the correlation of adjacent pixels in the plain-image so that neighbor pixels in the cipher-image virtually have no correlation. That is to say, the new scheme possesses prominent diffusion property.

### 5.3. Sensitivity Analysis

From the cryptographical point of view, given two distinct keys, even if their difference is the minimal value under the current finite precision, the encryption and decryption results of a good cryptosystem should still be completely different. In other words, this cryptosystem should have a very high sensitivity to the secret key [14]. For testing the key sensitivity of the proposed block encryption procedure, we use the grayscale image Lena.bmp of size 256 × 256 as the test image to illustrate the result and perform the following steps:

1. Lena.bmp is encrypted by using the secret key (*μ* = 3.998, *x_0_* = 0.21745) and the resultant image is referred as Ciphertext A;
2. The same image is encrypted by making the slight modification in the secret key *i.e.*, (*μ* = 3.998 + 10^−15^, *x_0_* = 0.21745) and the resultant image is referred as Ciphertext B;
3. Again, the same original image is encrypted by making the slight modification in the secret key *i.e.*, (*μ* = 3.998, *x_0_* = 0.21745 + 10^−15^) and the resultant image is referred as Ciphertext C;
4. The same original image is encrypted by making the slight modification in the secret key *i.e.*, (*μ* = 3.998, *x_0_* = 0.21745 − 10^−15^) and the resultant image is referred as Ciphertext D.
5. Finally, the correlation coefficients between the corresponding pixels of the four ciphertexts A, B, C and D are computed and filled into Table 7.

It is clear from the Table 7 that no correlation exists among four encrypted images even though these have been produced by using slightly different secret keys. These results sufficiently demonstrate the proposed cryptosystem is highly key sensitive.

**Figure 5.**
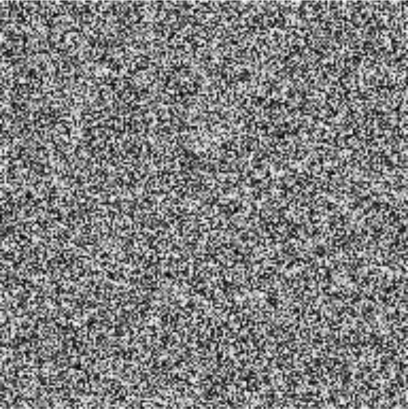
Bit-wise XOR of two ciphertexts.

Another cryptographical property required by a good cryptosystem is that the encryption should be very sensitive to plaintext, *i.e.*, the ciphertexts of two plaintexts with a slight difference should be very different [14]. Figure 5 is the bit-wise XOR of two ciphertexts when encrypting two image plaintexts with only the first bit different based on the proposed cryptosystem. The result of Figure 5 showing that the proposed encryption scheme is very sensitive with respect to small changes in the plaintext.

From the above investigation and study, we can conclude that the lack of security will discourage the use of these algorithms for secure applications. It is advisable that new chaotic cryptosystems take into account some important things: (1) the distribution of the ciphertext should be sufficiently flat in order to resist the statistics attack [8]; (2) the sub keys should depend on not only the secret key but also the plaintext to avoid key stream attack [11]; (3) the first block or sub key should be pretreated to resist some existing attacks; (4) the ciphertext should not leak out any information of the sub keys to eliminate corresponding utilizing ciphertext attacks.

## 6. Conclusions

This paper has analyzed the security of a block cipher based on logistic map proposed in [11]. It demonstrated that [11] is vulnerable to chosen ciphertext attack and key stream attack. Then it gave an enhancement version on wireless camera sensor network. Performance analysis demonstrates that the proposed scheme possesses the original benefits as well as enhancing its security. The sample procedure and efficiency of the new scheme are encouraging for the practical implementation in wireless camera sensor network.

## Figures and Tables

**Table 1.** Decryption of C1 using *μ* = 4, *x*_0_ = 0.1777.

*j*	*C_j_*	*f*(*C_j_*) mod 64	*D_j_*	*A_j_*
1	EAFA4D22D326D40C	35	10	5E0AEF19A566A729
2	2960D4C5E768138D	36	03	D6E5053AF966B07E
3	C716165410ACD847	12	1D	EF5FCAE1DB5FA883
4	3C991CA5F1E8FCC6	20	2E	4246A2AAADA975E2

**Table 2.** Decryption of C2 using *μ* = 4, *x*_0_ = 0.1777.

*j*	*C_j_*	*f*(*C_j_*) mod 64	*D_j_*	*A_j_*
1	F11ED8CA5F72155E	35	10	5E0AEF19A566A729
2	8A99683495FDBAAB	36	03	D6E5053AF966B07E
3	CC1E07D524E0E7A1	12	1D	EF5FCAE1DB5FA883
4	D9D58D603B600C1E	20	2E	4246A2AAADA975E2

**Table 3.** The chosen *C^1^* and *C^2^* of *C*.

*j*	*C_j_*	*f*(*C_j_*) mod 64	Chosen $C_{j}^{1}$	Chosen $C_{j}^{2}$
1	218A916626E5DA55	28	00000000000000DC	0000000000001C00
2	BA53340E52524733	45	00000000000000ED	0000000000002D00
3	2C2CE7EEB40BA7EC	63	00000000000000FF	0000000000003F00
4	B19F2A8A8BBAB8BD	62	00000000000000FE	0000000000003E00

**Table 4.** Decrypt the chosen *C^1^* and *C^2^* of Table 3 using *μ* = 4, *x*_0_ = 0.1777.

*j*	$P_{j}^{1}$	$P_{j}^{2}$	$P_{j}^{1}⊕P_{j}^{2}$	*D_j_*	*A_j_*
1	A7F55E0AEF19A566	BB295E0AEF19A566	1CDC000000000000	16	5E0AEF19A566A729
2	ABA16BD9AC1F83AC	ABB79D59AC1F83AC	0016F68000000000	25	B3583F075957423A
3	2B876D414E8FBD7F	2B8762BE8E8FBD7F	00000FFFC0000000	34	3A3EF5FCAE1DB5FA
4	6A2A9568E24C2424	6A252AE8E24C2424	000FBF8000000000	26	A389309091A8AAAB

**Table 5.** Entropy test result.

Test file	Lena	Text file	Video file
Ciphertext entropy	7.9923	7.9981	7.9919

**Table 6.** The correlation coefficients of the adjacent pixels.

**Positions**	**Plaintext image**	**Ciphertext image**
Horizontal	0.98448	0.0031261
Vertical	0.94878	0.0057563
Diagonal	0.96787	0.0130690

**Table 7.** The correlation coefficients of the ciphertexts.

**Ciphertext 1**	**Ciphertext 2**	**Correlation Coefficient**
Ciphertext A	Ciphertext B	0.00296
Ciphertext A	Ciphertext C	0.00137
Ciphertext A	Ciphertext D	0.00429
Ciphertext B	Ciphertext C	0.00153
Ciphertext B	Ciphertext D	0.00194
Ciphertext C	Ciphertext D	0.00296
